# Defective Homocysteine Metabolism: Potential Implications for Skeletal Muscle Malfunction

**DOI:** 10.3390/ijms140715074

**Published:** 2013-07-18

**Authors:** Sudhakar Veeranki, Suresh C. Tyagi

**Affiliations:** Department of Physiology and Biophysics, University of Louisville School of Medicine, Louisville, KY 40202, USA

**Keywords:** hyperhomocysteinemia, homocysteine, inflammation, muscle, dystrophy, degeneration, ROS, GPCR, NO, ER stress

## Abstract

Hyperhomocysteinemia (HHcy) is a systemic medical condition and has been attributed to multi-organ pathologies. Genetic, nutritional, hormonal, age and gender differences are involved in abnormal homocysteine (Hcy) metabolism that produces HHcy. Homocysteine is an intermediate for many key processes such as cellular methylation and cellular antioxidant potential and imbalances in Hcy production and/or catabolism impacts gene expression and cell signaling including GPCR signaling. Furthermore, HHcy might damage the vagus nerve and superior cervical ganglion and affects various GPCR functions; therefore it can impair both the parasympathetic and sympathetic regulation in the blood vessels of skeletal muscle and affect long-term muscle function. Understanding cellular targets of Hcy during HHcy in different contexts and its role either as a primary risk factor or as an aggravator of certain disease conditions would provide better interventions. In this review we have provided recent Hcy mediated mechanistic insights into different diseases and presented potential implications in the context of reduced muscle function and integrity. Overall, the impact of HHcy in various skeletal muscle malfunctions is underappreciated; future studies in this area will provide deeper insights and improve our understanding of the association between HHcy and diminished physical function.

## 1. Introduction

**Figure 1:**
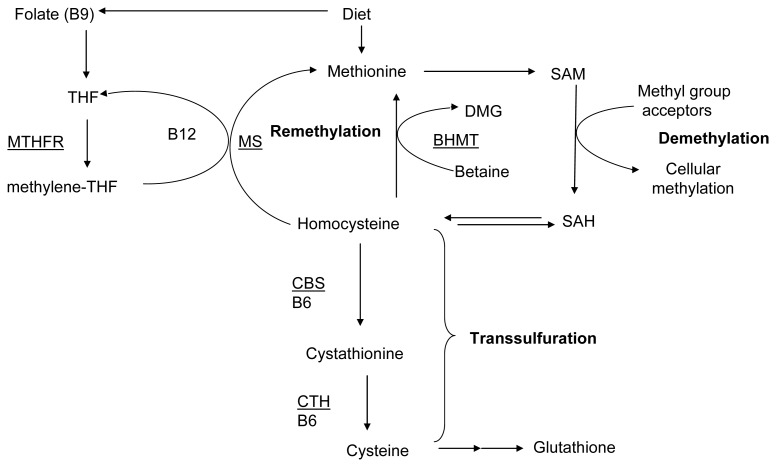
Schematic diagram of summarized homocysteine metabolism with key enzymes. THF (tetrahydrofolate); MTHFR (methylene tetrahydrofolate reductase); MS (methionine synthase); SAM (*S*-adenosylmethionine); SAH *(S*-adenosyl-homocysteine); CBS (cystathionine β-synthase); CTH (cystathionine γ-lyase). The enzymes are underlined. Another remethylation pathway involving betaine:homocysteine *S*-methyltransferase (BHMT) occurs only in liver and kidneys [3].

Hyperhomocysteinemia (HHcy) is a metabolic systemic disorder with defects in sulphur-containing amino acid (methionine and cysteine) metabolism leading to abnormally higher amounts of non-building-block intermediary amino acid homocysteine (Hcy). Genetic, nutritional and hormonal etiologies as well as age- and sex-mediated differences are identified in abnormal accumulation of homocysteine. HHcy leads to multi-organ failure including the brain, kidney, heart, vascular system and musculoskeletal system [1–3]. Normal levels of Hcy in the blood range from 10 to 12 μM and in very severe cases the concentrations might shoot above 100 μM, which leads to homocystinuria. Homocysteine is synthesized from methionine (Figure 1), absorbed from the digestive system, by a process called demethylation that involves the generation of *S*-adenosylmethionine (SAM) and *S*-adenosyl-homocysteine (SAH) as key intermediaries. Homocysteine is normally removed by two key processes: (1) the methionine cycle that synthesizes methionine from the Hcy utilizing *N*-5-methyltetrahydrofolate or betaine (in liver and kidney) as methyl donors, and (2) irreversible transsulfuration that converts Hcy to cystathionine and eventually to cysteine. Genetic mutations in the enzymes, methylene tetrahydrofolate reductase (MTHFR) and cystathionine β-synthase (CBS), involved in these two key processes and nutritional deficiencies of vitamin co- factors (folate, B12 and B6) are the primary causes of hyperhomocysteinemia and homocystinuria [4]. The molecular mechanisms underlying the homocysteine induced-pathology are currently under intense investigation.

In the recent past it has been observed that hyperhomocysteinemia is associated with diminished muscle function. The disrupted Z-discs and disorganized banding pattern along with excessive collagen deposition in the basal lamina were observed in a patient with homocystinuria [5]. Chronic administration of homocysteine has been shown to reduce rat skeletal muscle cell viability and produce energy imbalance [6]. Abnormally higher levels of homocysteine in both plasma and cerebrospinal fluids were found to correlate with amyotrophic lateral sclerosis (ALS), a motor neuronal disease that causes muscle degeneration [7,8]. Another neurological disorder that affects muscle function, multiple sclerosis, was also found to be associated with higher amounts of homocysteine in the plasma, especially in males [9]. Vascular inflammation, thrombosis and thrombo-embolism are the pronounced deleterious effects of hyperhomocysteinemia and results in peripheral arterial disease (PAD) [10] apart from other organ failure. PAD results in muscular damage, inflammation and loss of regeneration capability of muscles.

Aging studies revealed adverse effects of elevated homocysteine on the physical functions of older people [11]. Significant negative correlation was reported between the plasma Hcy levels and physical performance in elderly women [12,13]. Another large sample study with older people has also found that HHcy has been independently associated with declined physical function [14]. HHcy was shown to damage skeletal muscles as evidenced by increases in the muscle specific creatine phosphokinase isoform (CK) [15]. These results together with other studies [16] suggest that HHcy could deteriorate muscle function and integrity and might be responsible for the elderly frailty in a sub set of the cases. However, the mechanistic role of HHcy in declined muscle strength is unknown. The liver and kidneys mainly contribute to the Hcy levels in the plasma [3]. Nonetheless, it was demonstrated that the acute maximal anaerobic exercise, but not the short-term intense anaerobic exercise, raised the plasma Hcy levels [17,18],which can be alleviated by creatine supplementation. While strenuous exercise raised the Hcy plasma levels, the Hcy levels are near normal during the successive pre-season periods in the athletes [19,20]. Enhanced methylation demand might have a role in such elevations of Hcy during prolonged exercise [21]. Reduced plasma levels of vitamins involved in homocysteine metabolism (Figure 1) and chronic alcohol consumption might also contribute to HHcy [22]. In this review we have summarized the findings pertaining to various pathologies caused by hyperhomocysteinemia and outlined the putative mechanisms by which abnormally higher homocysteine levels might contribute to skeletal muscle malfunction.

Multiple hypotheses were evaluated to explain the HHcy induced pathophysiology in various organs: (1) reduced oxidative defense and enhanced production of reactive oxygen species (ROS); (2) alterations in gene expressions through epigenetic changes involving aberrant methylation; (3) inflammation and its associated changes; (4) inhibition of nitric oxide (NO) signaling; (5) enhanced endoplasmic reticulum (ER) stress; and (6) changes in the key signaling pathways. It is possible that Hcy may produce multiple changes listed above simultaneously when accumulated abnormally. Tissue composition, Hcy uptake, relative abundance of various Hcy metabolic enzymes and the duration of Hcy exposure in various organs determine the extent of damage caused by hyperhomocysteinemia. Here we have discussed the above mentioned HHcy mediated molecular changes and consequent potential implications in the context of muscle function and integrity. We have considered the effects of HHcy in vascular, neuronal, and myocyte function leading to impaired muscle function.

## 2. HHcy & Compromised Antioxidant Capability

Human muscles contain significant levels of cystathionine β-synthase (CBS) [23], which is involved in the first rate-limiting step of the transsulfuration process where cysteine is synthesized from homocysteine. Higher frequencies of mutations in this gene were reported which cause hyperhomocysteinemia and also cysteine deficiency to some extent [24]. Given that cysteine is indispensible for the synthesis of GSH, lack of cysteine uptake by the cells and/or deficiencies in transsulfuration enzymes might compromise the cellular antioxidant potential and may result in deleterious muscle pathologies. Cysteine uptake into cells is mediated through various amino acid transporters in the presence or absence of sodium [25,26]. It was demonstrated that homocysteine shares the cysteine transporters with a varying degree of affinity for it to enter into the cells [26,27]. In the cases of HHcy, competitive inhibition of cysteine entry into the cells might also compromise the cellular antioxidant potential, especially in tissues that lack enzymes for the transsulfuration process where cysteine uptake is the sole mechanism of the cysteine supply, such as cardiovascular tissues [26]. Furthermore, in the cases of mutant CBS and CST presence, HHcy could further aggravate the cellular oxidative stress by competitively inhibiting the cysteine uptake by the cells. Consistent with this hypothesis, dietary cysteine depletion in mice carrying deficient cystathionine γ-lyase (CTH) activity (another key enzyme in the transsulfuration process and also expressed in human skeletal muscles [23]) resulted in reduced glutathione content in skeletal muscles, enhanced sensitivity to oxidative stress, and lethal muscular atrophy [28]. Severe depletion of muscular glutathione levels, a major antioxidant in muscles, was also shown to compromise mitochondrial function and cause muscle degeneration [29]. Taken together, the levels of Hcy and cysteine could play a modulatory role in the determination of the extent of muscle damage during exercise under certain genetic predispositions.

Homocysteine was observed to stimulate intracellular superoxide radical production as well as superoxide dismutase (SOD) induction in endothelial cells (EC) [30]. It is also interesting to note from the experiments with endothelial cells in the presence of high amounts of Hcy, that Hcy is capable of NADPH oxidase (NOX-4) translocation into mitochondria and reduction in thioredoxin expression. These events are associated with enhanced ROS production that can be ameliorated by the activation of peroxisome proliferation activator receptor gamma (PPARγ) [31]. Furthermore, induction of NOX-4 and consequent ROS accumulation was also implicated in the fibroblast activation and secretion of inflammatory mediators [32]. It will be interesting to know if the reversal of abnormal homocysteine metabolism could reduce the pathological events caused by the compromised cellular antioxidant potential. ROS can have multiple targets in any given time based on the site and amount of its production. Hence, relevance of Hcy-mediated changes via ROS production and consequent ramifications in muscle malfunction need to be studied to understand muscle weakness associated with HHcy.

## 3. HHcy & Hypomethylation

Epigenetic events associated with DNA are reversible events but can stably transmit gene regulation information to daughter cells. SAM is the key methyl donor for cellular methylation events including DNA methylation, and this process produces SAH. SAH is converted to Hcy in a reversible reaction, and SAH levels were found to be elevated in patients with CBS mutations [1]. Elevated levels of SAH, and concomitant increases in the SAH/SAM ratio were proposed to inhibit cellular methylation reactions [1,33,34]. Consistent with this hypothesis, reduced Ras methylation and cyclin A transcription were observed. These events are associated with growth suppression in endothelial cells [33]. However, much higher doses of Hcy have been shown to induce cyclin A levels in vascular smooth muscle cells [35]. This apparent discrepancy could be due to different doses of Hcy, which may target different regulators and/or the same molecules differentially in a dose-dependent fashion. Alternatively, different cell types could respond differently owing to their differential gene expression. Furthermore, in contrast to the traditional view that DNA methylation is involved in the transcriptional silencing, it was demonstrated that DNA methylation cycles or presence of certain factors that bind to methylated DNA may result in transcriptional activation. [36–38]. Hence it is necessary to carefully evaluate the consequences of DNA methylation or lack of methylation in a context-dependent manner. It has been proposed that Hcy induced changes in methylation could also contribute to chromatin remodeling by recruiting methyl cytosine binding protein (MBP) and histone deacetylase (HDAC) to the CpG islands [33].

Changes in methylation also affect skeletal muscle remodeling and/or repair. Studies showed that Notch-1, a key signaling pathway involved in muscle regeneration, was inhibited by DNA methylation through NF-κB (nuclear factor kappa-light-chain-enhancer of activated B cells) activation of TNF-α (tumor necrosis factor) signaling [39]. Given such significance for DNA methylation in muscular dystrophy, it is of interest to ask whether defective Hcy metabolism modulates TNF-α mediated Notch-1 inhibition and regulates muscle regeneration in HHcy mediated loss of muscle endurance. It is plausible that defective Hcy metabolism might enhance SAH levels and SAH/SAM ratios and might produce methylation changes as in other tissues [34]. Understanding global changes in gene expression owing to altered DNA methylation in the presence of disease relevant doses of Hcy would provide a framework for comprehensive delineation of HHcy mediated skeletal muscle damage.

Changes in gene expression also involve microRNA expression variations apart from mRNA alterations. MicroRNAs (miRNA) are very small regulatory RNA of 18–22 bp and are mainly involved in the posttranscriptional control of target mRNA by pairing to complimentary sequences. Binding of miRNA to the target mRNA leads to either destabilization of the target mRNA or inhibition of translation. Both of these events eventually lead to the down regulation of targeted protein quantity and produce distinct changes in the cell signaling and behavior. Recent findings revealed a significant contribution of miRNA in regulation of muscle physiology and pathology [40,41]. For example, miR-133a family microRNA deletion, in mice, leads to adult onset centronuclear myopathy. In the absence of miR-133a, dynamin-2 is misregulated and produces mitochondrial dysfunction and altered muscle structure [42]. In another study, miR-499c was shown to induce myofibril formation and regulated cardiac marker expression [43]. It was shown that muscle-specific microRNAs, miR-1, miR-133 are downregulated in dystrophic muscle [44]. Downregulation of miR-1 and miR-29 are implicated in oxidative stress and fibrosis respectively [44]. Taken together, these studies imply that crucial microRNA downregulation is involved in muscular dysfunction and degeneration. As HHcy could cause gene expression changes, it is of interest to study if HHcy is involved in reduction of any of these crucial microRNAs. As such, delineation of changes in global organ/tissue/cell specific microRNA profiling in the presence of HHcy would aid in unraveling the molecular mechanisms of Hcy-mediated pathology and development of novel small molecule therapies.

The liver contributes to significant plasma levels of both Hcy and creatine [3,45]. Continuous supply of creatine is important for the integrity of the skeletal muscles and often determines the extent of endurance during exercise [46]. Synthesis of creatine consumes large amounts of methyl groups, which are supplied by the SAM [21]. It was demonstrated that CBS deficiency leads to accumulation of SAH, which reduces overall DNA methylation reactions in the liver [47]. However the impact of elevated SAH levels on the synthesis of creatine is not assessed. Genetic mutations that lead to HHcy may also elevate the SAH as Hcy can be converted to SAH in a reversible reaction (Figure 1). Given that SAH can inhibit the cellular methylation reactions [1], HHcy may potentially limit the liver supply of creatine and may reduce muscle endurance and enhance muscle damage.

## 4. HHcy & Inflammation

Inflammatory conditions associated with muscle cells are known as myositis and involve immune cell infiltration with varying degrees of protein aggregations and auto immune responses, which lead to degeneration, muscle weakness and fibrosis [48]. It was suggested that cytokine production (IL-1β, Interleukin-1 beta) in response to pathogens and other unknown factors could trigger a cascade of events involving NF-κB activation, ER stress, protein aggregation and immune cell infiltration to culminate in muscle cell necrosis and degeneration. HHcy has the potential to initiate and/or aggravate inflammation and cytokine production. Consistent with this hypothesis it was noted from the experiments with kidney podocytes that HHcy activates Caspase-1 through Nod-like receptor protein 3 (NLRP3) mediated inflammasomes, and mediates IL-1β secretion [49]. Interestingly, HHcy was reported to raise inflammatory mediators in both blood and in the tissues studied [50,51]. However, systemic inflammatory conditions such as sepsis are not associated with the Hcy elevations, implying that HHcy could be a causative factor for inflammation but may not be the consequence of inflammation [52]. Based on these studies, it is important to ask whether Hcy could similarly activate NLRP-3 based inflammasomes and cause IL-1β secretion in other tissues and cells as well. The outcomes from such studies not only enhance our knowledge with regard to the HHcy pathology, but also enable us to design new therapeutic interventions. HHcy could be a potential causative factor in inflammatory myositis of unknown etiology.

HHcy has also been shown to aggravate Angiotensin-II mediated inflammatory effects such as inflammatory cell infiltration into tunica adventitia of arteries, and elevation of interlukin-6 (IL-6) and monocyte chemoattractant protein-1 (MCP-1) levels in the plasma of mice bearing homozygous apolipoprotein E (ApoE) deletions in a model of Abdominal aortic aneurysm (AAA). Studies involving monocyte responses to Hcy also noted enhanced pro inflammatory cytokine secretion [53]. These proinflammatory molecules are instrumental in recruitment of inflammatory immune cells. Enhanced ROS (reactive oxygen species) and SMAD (small mothers against decapentaplegic) activation were proposed to mediate the proinflammatory effects of Hcy [32]. Future studies addressing the intricate relationship between enhanced ROS and SMAD activation in response to Hcy would provide mechanistic insights of Hcy-mediated inflammation. In this context it will be interesting to know if Hcy mediates SMAD activation and prevents muscle regeneration by promoting muscle inflammation.

## 5. HHcy & NO

Muscular endurance and adaptability to various external stimuli depends on efficient blood flow regulation and vascular system integrity in the muscle. The blood flow to muscle cells is typically regulated by nitric oxide (NO), which is synthesized from L-arginine by nitric oxide synthase (NOS). Mislocalization of NOS from sarcolemma and defective NO production were reported in several forms of muscular dystrophies and implicated in focal ischemia, diminished exercise endurance and fatigue [54]. Mice lacking nNOS (neuronal NOS), also anchored to sarcolemma, exhibited a lower capillary to fiber ratio and decreased vascular endothelial growth factor (VEGF) expression [55] and highlight both autocrine and paracrine capabilities of NO.

Hcy diminishes the bioavailability of NO through uncoupling of NOS and reduced uptake of arginine by the cells. Both of these mechanisms enhance ROS production. In the presence of elevated ROS, NO reacts with ROS and generates peroxynitrite limiting NO signaling [4]. Hence it is conceivable that excess Hcy might compromise NO signaling and limit hemodynamics in muscular vessels and result in fatigue, ischemia and reduced physical endurance. Support for this hypothesis can be seen in older people who exhibit higher levels of Hcy and less endurance to exercise [56]. Furthermore, hypertension and diabetes mellitus enhance Hcy levels and further compound the age related affects on muscles [57].

The dynamic supply of nutrients to skeletal muscles is supported by the demand-induced conductance of vasodilatation (CVD) from distal arteriole to proximal arterioles and feeding arteries. It has been shown that gap junctions comprised of connexins (Cx 37, 40 and 43) between arterial endothelial cells are important in carrying the hyperpolarization signals in an efficient ascending fashion and produce vasodilatation which mediates greater nutrient fetch to the skeletal muscles [58,59]. Recently it was reported that HHcy reduces expression of connexins in the skeletal muscle vascular system and perturbs CVD and tissue perfusion [60]. These findings suggest that in the cases of HHcy there is enhanced fatigability owing to reduced CVD and possibly explain why older people also have higher Hcy levels and less exercise endurance. How HHcy regulates connexin expression is currently unknown.

## 6. HHcy and Endoplasmic Reticulum (ER) Stress

The ER plays a pivotal role in proper assisted protein folding and posttranslational modifications of proteins for appropriate function, membrane targeting and secretion. Any process that interferes with ER function results in unfolded protein response (UPR) and ER stress wherein certain chaperonins (Bip/GRP78; glucose-regulated protein 78) and other proteins are transcriptionally induced to restore the ER function. Persistent unresolved ER stress subsequently activates endoplasmic reticulum-associated degradation (ERAD). Failure of UPR and ERAD eventually leads to toxic protein accumulation and compromises cellular function.

ER stress is important in skeletal muscle homeostasis, and abnormal ER stress leads to muscle diseases [61,62]. ER stress was suggested to enhance the risk of sporadic inclusion body myositis (sIBM), a disease of older persons with unknown etiology associated with intra-muscle fiber protein aggregates [63]. ER stress was also proposed to derail autophagy, which leads to protein aggregates. Given that older persons tend to have higher levels of Hcy and that HHcy was shown to induce ER stress, it is worthwhile to know if HHcy could initiate or aggravate sIBM.

HHCy was shown to induce Bip, a marker of ER stress, along with other genes in endothelial cells and also alters protein secretion [64–67]. Two hypotheses can explain HHcy-induced ER stress: (1) enhanced ROS production and compromised antioxidant potential [68]; and (2) homocysteinylation of resident ER chaperon protein Bip [69]. Whether HHcy affects the ER function to cause diminished muscle performance is currently unknown.

## 7. HHcy & Cell Signaling Pathways

### 7.1. TGF-β Signaling

Recent studies showed that TGF-β (transforming growth factor) signaling suppresses muscle regeneration. Mutations in the muscle extracellular matrix protein, fibrillin-1, could cause excessive activation of TGF-β signaling, which might be responsible for diminished muscle regeneration and muscle pathologies associated with multiple human myopathies [70]. Hyperhomocysteinemia causes excess induction of TGF-β and changes in extracellular matrix (ECM) regulators (matrix metalloproteinases (MMP) and Tissue inhibitors of metalloproteinases (TIMP), which have been observed in cardiac muscle [71]. Furthermore, the concurrent cysteine deficiency associated with hyperhomocysteinemia was shown to deplete the levels of extracellular microfibril, fibrillin-1 [24] and might compromise muscle regeneration through excessive TGF-β signaling. It was also shown that losartan, an angiotensin-II type 1 receptor (AT-1) blocker that suppresses TGF-β signaling, could rejuvenate the muscle regeneration potential [70]. Broader understanding of the impact of this drug on the hyperhomocysteinemia induced muscle dysfunction would provide additional therapeutic options.

Homocysteine was also shown to induce the connective tissue growth factor (CTGF), which is involved in fibrosis of vascular smooth muscle cells [72]. However, it is unknown whether the induction of this gene by homocysteine also contributes to skeletal muscle fibrosis. Furthermore, unraveling the mechanisms by which homocysteine induces CTGF would provide targets for intervention. Given that hyperhomocysteinemia also leads to muscular abnormities such as muscle degeneration and fibrosis, it is possible that chronic excess Hcy levels might alter the muscle regenerative potential through abnormal TGF-β signaling and misbalancing the ECM regulators.

### 7.2. GPCR (G-protein Coupled Receptor) Signaling

Research involving vascular smooth muscle cells (VSMC) has discovered that Hcy potentiates Angiotensin II induced Ca^2+^ release, upon activation of Angiotensin II type I receptor (Gα_q_βγ type of GPCR), mainly from the intracellular Ca^2+^ stores [73]. These effects are highly specific to homocysteine as the analogous thiol-containing major amino acid, cysteine, could not induce Ca^2+^ releases. The effects of Hcy on Ca^2+^ release in the VSMC can be blocked by inhibiting the protein tyrosine kinases (PTK), inosito1,4,5-trisphosphate (IP3) and G protein coupled receptors (GPCR) [73]. These findings suggested modulation of GPCR signaling by Hcy. In the case of microvascular endothelial cells (MVEC), Gα_i_ activation by HHcy is proposed to mediate Ca^2+^ and PTK-dependent, ERK-driven induction of MMP9, which is implicated in Hcy associated pathologies in cardiovascular diseases [74]. However, the molecular mechanisms of HHcy induced GPCR activation remains to be solved. Nonetheless, Hcy has also been reported to modulate activation dependent down regulation of multiple sub-families of GPCRs which include Gα_q_, Gα_i_, and Gα_12/13_[75].

Angiotensin II type I receptor (AT-1) activation by its ligand, Angiotensin II, is implicated in skeletal muscle fibrosis [76,77]. Activation of AT-1 leads to enhanced ROS production, p38 MAPK (mitogen activated protein kinase) activity and TGF-β expression in the muscle cells. Furthermore, ablation of TGF-β led to decreased fibronectin (FN) and CTGF expression, which were up regulated by Angiotensin II consistent with its pro fibrotic effects [76]. When considered HHcy also modulates GPCR function, it is conceivable that HHcy might aggravate angiotensin II effects on the skeletal muscles, and chronic exposure might cause fibrosis and reduced muscle regeneration. The evaluation of effects of HHcy on muscle either independently or in combination with Angiotensin II would help in understanding the lack of muscular endurance during old age and fibrotic conditions in different muscular dystrophies. Furthermore, it would be interesting to see the dynamics of Gα_q_ in the presence of both Angiotensin II and HHcy in muscle cells.

Another interface between HHcy and regulation of GPCR comes from the studies involving cardiac hypertrophy in a rat model of abdominal aortal constriction. Changes in β-adrenergic receptors (β-AR), Gα_s_ type of GPCR, mediate cardiac responsiveness to catecholamines, and often determine the degree of cardiac contractility in response to sympathetic tone. It was shown that SAH, a potent inhibitor of cellular methylation which accumulates during HHcy, significantly attenuates SAM mediated enrichment of phosphatidylcholine (PC) in the membranes which results in enhancement in the number of β-AR present [78] in the membrane. Consistent with these observations, another study has shown that diabetes mellitus associated HHcy was correlated with down regulation of β2-AR, and exercise improved β2-AR responses and reduced association between Hcy and β2-AR [79]. However, it is unknown whether decreased Hcy levels or changes in the β2-AR levels and/or modifications are responsible for the observed reduction in association between Hcy and β2-AR after exercise in diabetic mice.

Age dependent responsiveness of β-AR in the skeletal muscle is rather controversial [80]. The confounding factors such as diabetes, Hcy levels, and other biochemical parameters/health conditions might be responsible for such variations. Nonetheless, diminished β-AR responses are implicated in age dependent sarcopenia, reduced vascularity and enhanced fatigability [80]. It is important to know if there are any HHcy associated changes in β-AR responsiveness in skeletal muscles from the perspective of healthy aging.

**Figure 2:**
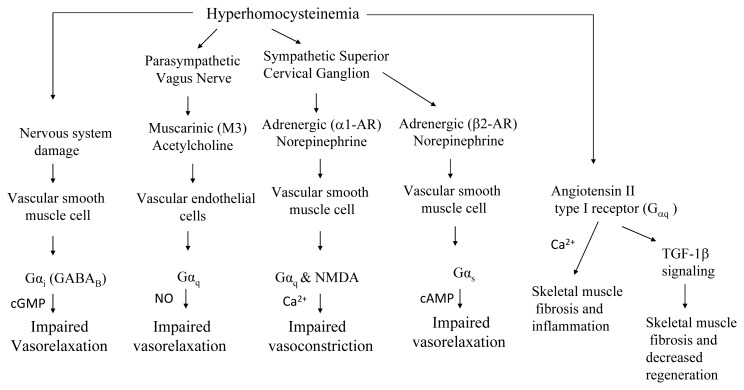
The HHcy modulation of sympathetic and parasympathetic nervous system and GPCRs are presented in the schematic diagram. Such modulation is observed to cause impairment in vascular responses to the regulatory signals. HHcy might damage the nervous system involving both parasympathetic and sympathetic system and disrupt autonomous regulation of hemodynamics [63]. Under normal circumstances, sympathetic stimulation activates either adrenergic α1-AR or β2-AR, which is Gα_q_ or Gα_s_ type of GPCRs respectively in the vascular smooth muscle. Agonist (Norepinephrine) binding to the α1-AR leads to the activation of phospholipase C through Gα_q_. This process eventually leads to the synthesis of second messengers Inositol triphosphate (IP3) and diacylglycerol (DAG). IP3 further releases Ca^2+^ from the internal stores and eventually produces muscle contraction by activating myosin light chain kinase (MLCK) present in the smooth muscles [83,87]. In addition, NMDA receptors can also be activated in the presence of activated PKC and other Src tyrosine kinases and increases Ca^2+^ influxes from the cell exterior [88]. Binding of agonist (Norepinephrine) to β2-AR leads to activation of adenylyl cyclase and results in cAMP production. cAMP inhibits MLCK through PKA (protein kinase A) and produces vasorelaxation [89]. Activation of the vagus nerve stimulates muscarinic acetylcholine receptors in the vascular endothelial cells which results in increased release of Ca^2+^ ions to produce NO [90]. NO diffuses quickly and activates guanylyl cyclase (GC) in the smooth muscles in a paracrine signaling fashion. The consequent production of cGMP in the vascular smooth muscle cells mediates vascular relaxation. Activation of GABA_B_ receptors was also observed to cause vasorelaxation [60], however the second messenger is unknown. Probably cGMP might mediate vasorelaxation after GABA_B_ activation. Angiotensin II type I receptor activation was shown to enhance intracellular Ca^2+^ levels and TGF-1b signaling, both of which could contribute to reduced muscle regeneration and enhanced muscle fibrosis. HHcy was shown in aberrant activation of Angiotensin II receptor [47,76,91,92].

Impaired vascular reflexes including constriction and relaxation have been proposed to mediate HHcy induced vascular pathology (Figure 2). In VSMCs, GPCR signaling induced second messengers; cGMP & cAMP (cyclic guanosine monophosphate and cyclic adenosine monophosphate) and Ca^2+^ are involved in the vasorelaxation and vasoconstriction respectively [81–83]. GABA_B_ (gamma-aminobutyric acid) has also been reported to cause vascular relaxation [84]. Parasympathetic stimulation enhances NO production in the vascular endothelial cells and contributes to vascular relaxation [85,86]. Given that Hcy can modulate GPCR signaling by direct binding; it is conceivable that during HHcy these vascular reflexes are impaired. Furthermore, HHcy has been shown to damage neurons as evidenced by upregulation of neuronal specific creatine kinase isoenzymes (CK BB) [15]. When considered that both parasympathetic (Vagus nerve) and sympathetic (superior cervical ganglion) nervous system can be damaged by HHcy, both of these non-functional autonomous tones may also impair vasorelaxation and vasoconstriction via aberrant GPCR signaling (Figure 2) in HHcy. These impaired vascular reflexes might in turn yield muscular pathologies such as degeneration, impaired tissue perfusion and compromised muscular function.

## 8. Conclusions and Future Perspectives

Homocysteine lowering trials using combinatorial vitamin therapy did not observe improvements in clinical conditions related to cardiovascular and neurological disorders [1]. The reasons for such failures could be multifactorial. The stage of treatment, age of patients and presence of other disease conditions such as diabetes might have influenced the outcome. The vitamin therapies could only have reversed the Hcy levels but were unable to restore the cellular homeostasis distorted by HHcy. Furthermore, prevention strategies would have given better outcomes rather than strategies aimed at lowering the Hcy levels at the end stage of diseases. In this context it is worthwhile to distinguish the reversible Hcy cellular/tissue changes from the irreversible ones. Studies aimed at unraveling the delicate changes caused by the chronic mild to moderate HHcy levels before full onset of the disease conditions would hold the key to better interventions.

**Figure 3:**
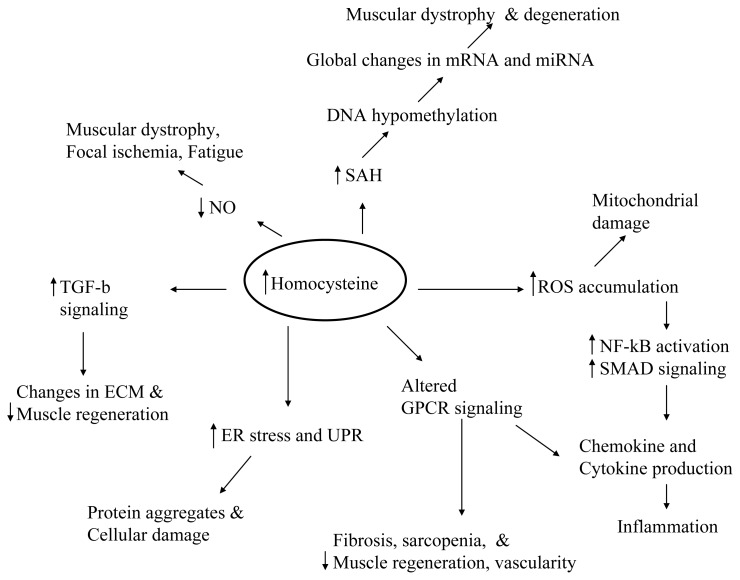
Schematic diagram showing the putative HHcy mechanisms in muscle pathology. It is possible that HHcy might disrupt several of these targets simultaneously.

The impact of HHcy over different cell signaling pathways including GPCRs and consequent cellular outcomes are increasingly realized. GPCRs are very important in modulation of cell structure, cell microenvironment, and cell responses to various stimuli. GPCR targeting has been involved in the regulation of vascular as well as muscular function and both the vascular and muscular integrity determines the long-term muscle performance. HHcy by its role in GPCR function modulation might have profound influence over skeletal muscle function (Figures 2 and 3) as in the cardiovascular system.

It is possible that Hcy, while having its own influence over different cellular processes, might produce synergistic effects in certain disease/genetic/altered systemic conditions. In the case of muscle specific disease processes, HHcy’s role as aggravator is apparently evident owing to Hcy influence over key processes such as oxidative stress, NO signaling, ER stress, methylation and gene expression, altering cell signaling and initiation of inflammation (Figure 3). Further evaluation of these processes in the muscle physiology with HHcy mimicking conditions is warranted before derivation of any firm conclusions. Development of appropriate disease models is the key for success of such studies. There are specific differences with regard to the extent of gene expressions in the human and mouse skeletal muscles. For example, CBS and CTH are present in much higher amounts in human muscles than in mouse muscles [23]. Hence mutations in these genes might possibly have a profound influence in the human disease development. It would be interesting to see if any of the gene mutations that elevate Hcy are associated with the gene mutations that cause loss of muscle function. The evaluation of HHcy’s role in muscle specific disease processes such as degeneration, dystrophies, compromised regeneration, inflammatory myopathies, fibrosis, reduced endurance and function will open new avenues for development of more efficient therapeutic strategies.
